# Perioperative nutritional support is associated with attenuated early postoperative albumin decline after gastrectomy for gastric cancer: a retrospective cohort study and machine learning prediction model

**DOI:** 10.3389/fmed.2026.1843582

**Published:** 2026-07-08

**Authors:** Tao Wen, Xikui Zhang, Xiaoping Zhang, Sumei Lv, Liman Yang, Bibo Tan, Feng Xia Liu

**Affiliations:** 1 Department of Operating Room, The Fourth Hospital of Hebei Medical University, Shijiazhuang, Hebei, China; 2 Department of Ophthalmology, The Fourth Hospital of Hebei Medical University, Shijiazhuang, Hebei, China; 3 Department of Gastrointestinal Surgery, The Fourth Hospital of Hebei Medical University, Shijiazhuang, Hebei, China; 4 Department of Nursing, The Fourth Hospital of Hebei Medical University, Shijiazhuang, Hebei, China

**Keywords:** gastrectomy, gastric cancer, machine learning, malnutrition, perioperative nutritional support, prediction model, serum albumin

## Abstract

**Introduction:**

Perioperative nutritional support is recommended for surgical patients at nutritional risk, but its relationship with early postoperative serum albumin recovery after gastrectomy for gastric cancer remains uncertain, and clinicians lack practical tools to identify patients most likely to show early albumin improvement.

**Methods:**

We conducted a retrospective cohort study of 1,529 adults who underwent gastrectomy for gastric cancer at The Fourth Hospital of Hebei Medical University between January and December 2017 and had both preoperative and early postoperative albumin measurements. Perioperative nutritional support was defined using the binary classification available in the source dataset. The primary outcome was postoperative albumin improvement, defined as postoperative albumin exceeding the preoperative value. Secondary outcomes were postoperative total protein improvement and change in albumin (postoperative minus preoperative). Associations were assessed using multivariable linear and logistic regression, and a gradient boosting model using 46 routinely collected preoperative and perioperative variables was developed to predict albumin improvement.

**Results:**

Albumin improved in 233 of 1,529 patients (15.2%) and was more common in the nutritional support group than in the no/standard support group (19.2% vs. 12.8%; *p* = 0.001). In adjusted analyses, nutritional support was associated with a higher *Δ* albumin (*β* = 0.572 g/L, 95% CI 0.111–1.033; *p* = 0.015), whereas the adjusted association with binary albumin improvement was imprecise (OR = 1.331, 95% CI 0.982–1.805; *p* = 0.065). The gradient boosting model achieved an AUROC of 0.711 and an AUPRC of 0.422 on the held-out test set.

**Discussion:**

Perioperative nutritional support was associated with an attenuated early postoperative decline in serum albumin after gastrectomy for gastric cancer, and the prediction model showed moderate discrimination for early albumin improvement. External validation and prospective evaluation are needed before clinical deployment.

## 1 Introduction

Gastric cancer remains a leading cause of cancer-related morbidity and mortality worldwide (1). Although advances in screening, perioperative management, and systemic therapies have improved outcomes in some regions, gastrectomy continues to be a cornerstone of curative-intent treatment for resectable disease. Surgical resection is accompanied by substantial metabolic stress, inflammatory activation, and short-term reductions in oral intake that may jointly compromise early postoperative recovery and increase vulnerability to complications.

Patients with gastric cancer frequently present with pre-existing malnutrition due to anorexia, early satiety, tumor-related obstruction, and cancer-associated inflammation. In addition, the perioperative period is characterized by catabolism, insulin resistance, and altered protein metabolism, which can further deplete lean body mass and impair immune competence and wound healing. Malnutrition has been consistently linked to adverse surgical outcomes, including infectious complications, delayed functional recovery, longer hospital stays, and increased healthcare costs (2, 3).

Accordingly, contemporary perioperative care pathways emphasize systematic nutritional screening and timely nutritional interventions. Guidelines from the European Society for Clinical Nutrition and Metabolism (ESPEN) and consensus criteria such as the Global Leadership Initiative on Malnutrition (GLIM) support a structured approach that includes (i) identification of nutritional risk, (ii) optimization of intake with oral nutritional supplements (ONS) where feasible, and (iii) escalation to enteral nutrition (EN) or parenteral nutrition (PN) when oral intake is insufficient. Enhanced recovery after surgery (ERAS) programs further promote early postoperative feeding and multimodal measures to reduce surgical stress and facilitate rehabilitation (2–5).

Despite broad agreement that nutritional support is important, the clinical effects of perioperative nutritional interventions in gastric cancer surgery appear heterogeneous. Randomized trials and meta-analyses have reported benefits for selected outcomes (e.g., body weight preservation, immune/inflammatory markers, or postoperative infections) for interventions such as ONS or immunonutrition, but effect sizes vary by patient selection, intervention composition, timing, and adherence. In routine clinical practice, nutritional support strategies are often individualized, and their impact on early biochemical recovery markers after gastrectomy remains incompletely understood (6, 7).

Serum albumin is routinely measured around the time of major surgery. Although frequently interpreted as a marker of nutritional status, albumin is also a negative acute-phase reactant and is influenced by inflammation, hemodilution, capillary leak, and perioperative fluid administration. For gastrointestinal surgery, early postoperative decreases in albumin (often summarized as ΔAlb, postoperative minus preoperative) have been associated with postoperative morbidity and may reflect the magnitude of surgical stress and the systemic inflammatory response. Whether perioperative nutritional support is associated with less severe early postoperative albumin decline or with an early “improvement” pattern (postoperative albumin exceeding the preoperative value) after gastrectomy has not been well characterized in large real-world cohorts (8–12).

From a clinical decision-making perspective, it is not sufficient to understand average associations: clinicians also need tools to identify which patients are most likely to show early biochemical recovery and, potentially, who may benefit from intensified nutritional strategies. Machine learning approaches can integrate multivariable perioperative information to estimate individualized probabilities of an outcome. In surgical oncology cohorts, gradient boosting decision tree models have demonstrated competitive performance for prediction tasks (13), but many published models suffer from limited reporting, restricted generalizability, and inadequate validation. Prediction modeling in this setting should therefore follow established guidance for transparent reporting and assessment of risk of bias (14–17).

In this retrospective cohort study, we pursued two complementary aims. First, we assessed the association between perioperative nutritional support and early postoperative albumin recovery indicators after gastrectomy for gastric cancer, focusing on both a continuous outcome (*Δ* albumin) and a clinically interpretable binary outcome (postoperative albumin improvement). Second, we developed and internally validated a gradient boosting prediction model for postoperative albumin improvement using routinely available preoperative and perioperative variables, with the goal of providing a practical foundation for future external validation and prospective evaluation.

## 2 Materials and methods

### 2.1 Study design and reporting standards

We performed a retrospective cohort study using routinely collected electronic medical record data from a tertiary referral center in China. Reporting follows the Strengthening the Reporting of Observational Studies in Epidemiology (STROBE) statement for observational studies (18) and the TRIPOD+AI guidance for prediction model development and validation (14).

### 2.2 Setting and data source

Data were obtained from the electronic medical record (EMR) and perioperative clinical information systems of The Fourth Hospital of Hebei Medical University, a tertiary referral center in Shijiazhuang, Hebei Province, China. The study period was January 1, 2017 to December 31, 2017. Variables were extracted from structured fields whenever available. For variables captured only in free-text documentation (e.g., operative reports), two trained investigators abstracted data using a standardized form; disagreements were resolved by consensus with a third reviewer, and a random 10% sample was double-abstracted for quality control. Laboratory measurements were reported in standard clinical units (albumin and total protein in g/L). The preoperative laboratory value was defined as the last measurement obtained within 7 days before surgery, and the early postoperative value was defined as the first measurement obtained on postoperative day 1–3 (within 72 h) after completion of surgery.

### 2.3 Participants

We included adult patients (≥18 years) who underwent curative-intent gastrectomy for gastric cancer and had both preoperative and early postoperative serum albumin values available to define the primary outcome. Gastrectomy procedures included distal, total, and proximal gastrectomy performed via open or minimally invasive approaches (laparoscopic or robotic), with reconstruction (e.g., Billroth I, Billroth II, Roux-en-Y, or esophagojejunostomy) determined by tumor characteristics and surgeon preference. We excluded patients undergoing emergency surgery, palliative procedures without tumor resection, concomitant major organ resections beyond standard gastrectomy (e.g., hepatectomy or pancreatectomy), re-operations during the index admission, and those with missing exposure information or missing preoperative or postoperative albumin measurements. A total of 1,529 patients met eligibility criteria and were included in the analytic cohort.

### 2.4 Ethics

The study protocol was approved by the Institutional Review Board/Ethics Committee of The Fourth Hospital of Hebei Medical University (approval No. 2025KS153). The requirement for written informed consent was waived because of the retrospective design and the use of de-identified routinely collected data, in accordance with local regulations.

### 2.5 Exposure: perioperative nutritional support

The exposure of interest was perioperative nutritional support, operationalized using a binary classification available in the source dataset (nutrition-support classification). Group 1 represented receipt of perioperative nutritional support, whereas Group 0 represented no or standard nutritional support. The binary label was derived from documented nutrition-support orders in the EMR and therefore reflected routine clinical practice rather than randomization or allocation by a study-specific protocol. The decision to prescribe perioperative nutritional support was made by the treating clinical team according to patient condition, expected intake, and local practice. Perioperative nutritional support was defined as any targeted nutritional support documented in the EMR order system beyond routine diet advancement, including oral nutritional supplements, enteral nutrition (tube feeding), and/or parenteral nutrition, initiated within 7 days before surgery and/or within postoperative day 7. Standard care (Group 0) comprised routine perioperative fasting and postoperative diet advancement as tolerated under the institutional enhanced recovery after surgery (ERAS) pathway without additional nutrition-support orders. Because the exposure was available only as a binary classification in the dataset, we did not further subclassify by modality, formulation (standard vs. immunonutrition), or caloric/protein dose in the primary analysis. No standardized caloric or protein targets were available in the source dataset; consequently, Group 1 should be interpreted as a heterogeneous exposure category rather than a single standardized regimen.

### 2.6 Outcomes

The primary outcome was postoperative albumin improvement, defined as a postoperative serum albumin value greater than the preoperative value (binary). This definition was chosen to identify patients who demonstrate an early biochemical recovery pattern despite the expected postoperative decline in albumin following major abdominal surgery. Clinically, this binary endpoint should be interpreted as a stringent and easily communicated indicator of a favorable early postoperative biochemical trajectory, rather than evidence of nutritional repletion. Secondary outcomes were (i) postoperative total protein improvement (postoperative total protein greater than the preoperative value; binary) and (ii) the change in albumin (*Δ* albumin, g/L), calculated as postoperative minus preoperative albumin. Throughout the manuscript, ‘albumin improvement’ refers to the binary endpoint, whereas ‘Δ albumin’ refers to the continuous postoperative-minus-preoperative difference. For the continuous outcome, extreme values were winsorized to reduce the influence of outliers as described in the underlying analysis report (at the 1st and 99th percentiles).

Because serum albumin is influenced by the surgical stress response and perioperative fluid shifts, it should be emphasized that albumin recovery endpoints in this study are intended as early physiological recovery indicators rather than definitive markers of nutritional status. In this dataset, concurrent inflammatory markers (e.g., C-reactive protein), perioperative fluid balance variables, and detailed postoperative complication data were not consistently available for inclusion; future prospective studies should collect these measures to better interpret albumin kinetics.

### 2.7 Candidate predictors for the prediction model

Candidate predictors (*n* = 46) were selected *a priori* from variables routinely available before or during the perioperative period to facilitate real-world applicability. To avoid information leakage, postoperative albumin, postoperative total protein, and derived outcome indicator variables were excluded from the predictor set. Predictors included demographics (sex, age, body mass index), comorbidities (e.g., hypertension, heart disease, diabetes), lifestyle factors (smoking and alcohol consumption), tumor characteristics and staging variables (tumor location, Borrmann type, T/N/M stage, AEG classification), preoperative treatment variables (e.g., neoadjuvant chemotherapy and treatment cycles), procedure-related indicators (e.g., hyperthermic lavage, HIPEC), and a panel of preoperative laboratory values (e.g., albumin, total protein, blood counts, liver enzymes, tumor markers). Body mass index (BMI) was calculated from weight and height.

### 2.8 Statistical analysis for association objectives

Categorical variables were summarized using counts and percentages. The proportions of patients with albumin improvement and total protein improvement were compared between nutritional support groups using the *χ*^2^ test. Risk differences with 95% confidence intervals were reported for unadjusted comparisons of binary outcomes.

For the continuous outcome (*Δ* albumin), we fitted a multivariable linear regression model with HC3 robust standard errors to account for potential heteroscedasticity. For the binary primary outcome (albumin improvement), we fitted a multivariable logistic regression model. The covariate adjustment set was prespecified based on clinical relevance and data availability and included age, sex, body mass index, comorbidities (hypertension, heart disease, and diabetes mellitus), tumor stage (T, N, and M stage), neoadjuvant chemotherapy (yes/no), and baseline preoperative albumin. For the *Δ* albumin model, we additionally adjusted for baseline preoperative total protein and preoperative laboratory markers reflecting hematologic status and physiologic reserve (white blood cell count, neutrophil count, lymphocyte count, hemoglobin, platelet count) and hepatic function (alanine aminotransferase, aspartate aminotransferase, and total bilirubin). Missing data were handled using complete-case analysis for each model; thus the multivariable linear regression was conducted in the available complete-case sample (*n* = 1,297). Missingness was concentrated in the expanded preoperative laboratory covariate set used for the *Δ* albumin model: 232 of 1,529 patients (15.2%) had at least one missing value among these additional covariates. The multivariable logistic regression was conducted in the full cohort (*n* = 1,529) because the core adjustment covariates had no missing values; no imputation was performed. Complete-case analysis was chosen because missingness was moderate and primarily involved auxiliary laboratory covariates, but this approach may introduce bias if missingness was related to unmeasured illness severity, clinical workflow, or treatment pathways.

Effect estimates are presented as regression coefficients (*β*) for *Δ* albumin and odds ratios (ORs) for albumin improvement, each with 95% confidence intervals. Two-sided *p* values were reported with a nominal significance threshold of 0.05. Given the observational design, results should be interpreted as associations rather than causal effects; confounding by indication remains possible.

### 2.9 Prediction model development and evaluation

We developed a gradient boosting decision tree model to predict postoperative albumin improvement using the 46 candidate predictors. The dataset was split into training and test sets using a stratified 80/20 split to preserve the outcome prevalence in both partitions. Within the training set, 5-fold cross-validation was used for model selection and hyperparameter tuning using a randomized search strategy. Tuned parameters included the number of boosting iterations (n_estimators/max_iter), learning rate, maximum tree depth, minimum samples per leaf, and row/feature subsampling fractions (when supported by the implementation). Because only 15.2% of patients showed albumin improvement, a model could appear accurate by mainly identifying non-improvers while still missing many true improvers. AUPRC was therefore prioritized because it summarizes precision (the proportion of predicted improvers who truly improved) and recall/sensitivity (the proportion of true improvers detected), directly reflecting performance for the minority positive class that is most relevant to clinical screening. The optimization metric for model selection was AUPRC, given the class imbalance. The final model was refit on the full training set using the optimal hyperparameters identified in cross-validation and then evaluated once on the held-out test set. This held-out set was drawn from the same single-center cohort and therefore represents internal validation rather than external validation.

Model discrimination was quantified using the area under the receiver operating characteristic curve (AUROC) and the area under the precision–recall curve (AUPRC). AUPRC was emphasized given the class imbalance, as precision–recall analysis is often more informative than AUROC when events are rare (19). In clinical terms, this emphasis aligns the model-selection criterion with the goal of finding the relatively small subgroup of patients predicted to show albumin improvement. Calibration was assessed using the Brier score (20). To facilitate clinical interpretability, a single operating point was selected using the Youden index (maximizing sensitivity + specificity − 1) (21), and we reported sensitivity, specificity, PPV, NPV, and accuracy at this threshold.

To characterize the relative contribution of predictors, permutation importance was computed using AUPRC as the scoring metric, and the top-ranked predictors are reported. Permutation importance provides a model-agnostic assessment of which variables most strongly influence predictive performance, but it does not establish causal relevance.

All analyses were conducted in Python (version 3.x) using standard scientific computing libraries including pandas, numpy, scipy, statsmodels (for regression with robust standard errors), scikit-learn (for model development, cross-validation, and performance evaluation), and matplotlib (for figures).

## 3 Results

### 3.1 Cohort composition and outcome frequency

**Figure 1:**
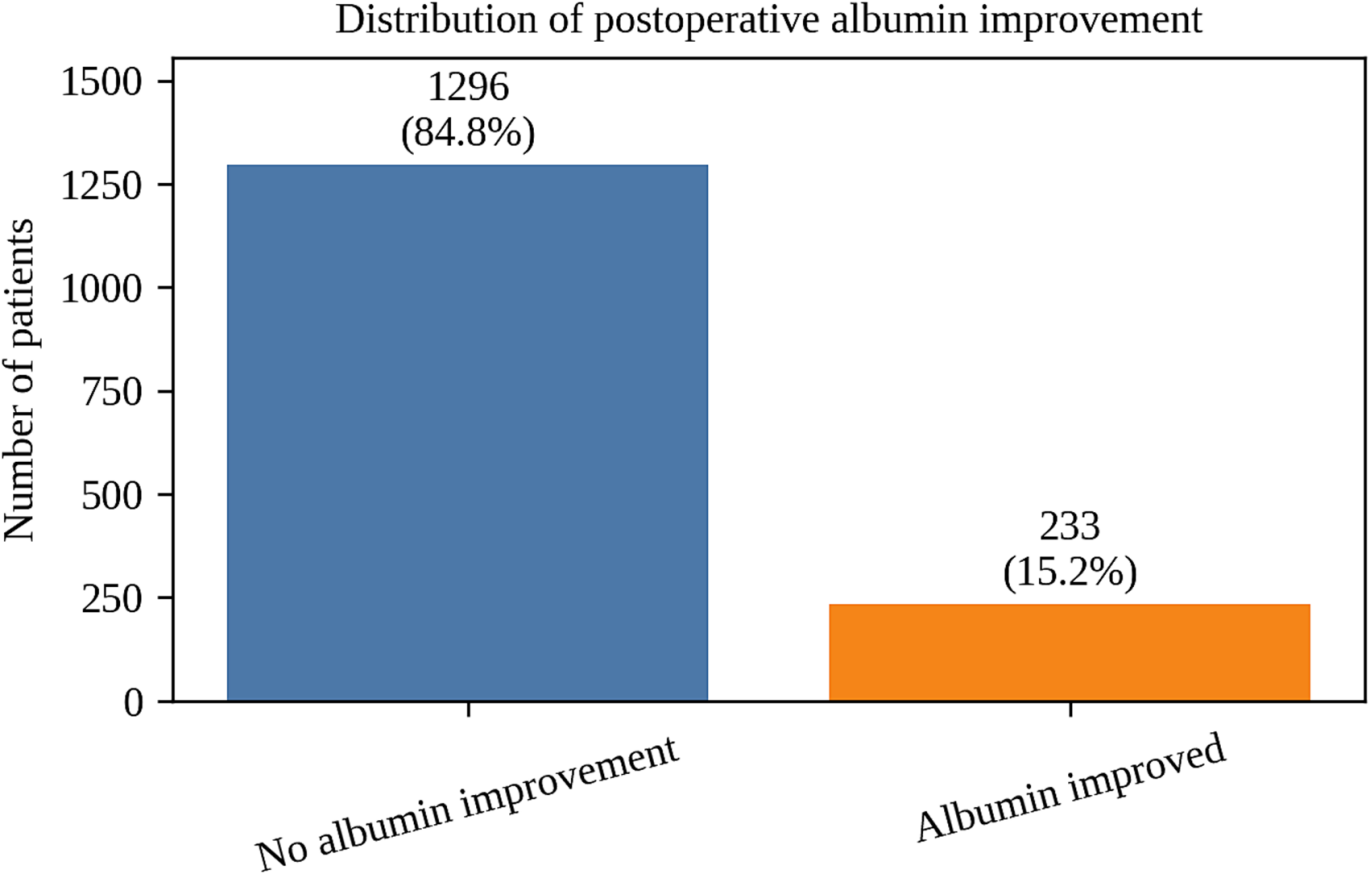
Distribution of postoperative albumin improvement in the overall cohort (*n* = 1,529).

The analytic cohort comprised 1,529 patients who underwent gastrectomy for gastric cancer. Of these, 939 (61.4%) were classified as receiving no or standard nutritional support (Group 0) and 590 (38.6%) were classified as receiving perioperative nutritional support (Group 1) (Table 1). Overall, 233 patients (15.2%) met the definition of postoperative albumin improvement (Figure 1). Postoperative total protein improvement occurred in 190 patients (12.4%).

**Table 1:** Cohort composition and unadjusted outcomes by perioperative nutritional support group.

Group	*N*	Albumin improved, *n* (%)	Total protein improved, *n* (%)
No/standard support (0)	939	120 (12.8)	109 (11.6)
Perioperative nutritional support (1)	590	113 (19.2)	81 (13.7)
Total	1,529	233 (15.2)	190 (12.4)

### 3.2 Unadjusted comparisons by nutritional support group

**Figure 2:**
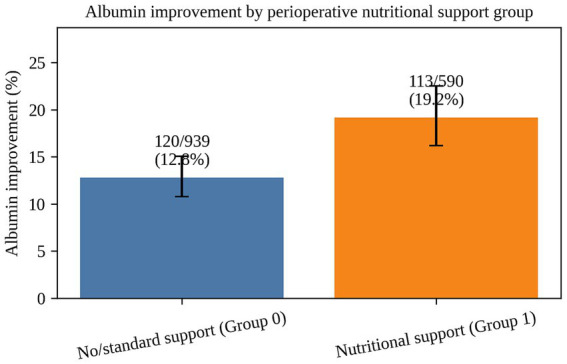
Postoperative albumin improvement by perioperative nutritional support group. Bars show group-specific proportions with 95% confidence intervals (Wilson method).

Albumin improvement was more frequent among patients who received perioperative nutritional support than among those who did not (19.2% vs. 12.8%; risk difference 6.4 percentage points, 95% CI 2.5–10.2; *p* = 0.001) (Table 1; Figure 2). In contrast, the proportion of patients with total protein improvement did not differ materially between groups (13.7% vs. 11.6%; *p* = 0.253). These unadjusted comparisons suggest a potential association between nutritional support and early albumin recovery, whereas total protein changes appeared less sensitive to the exposure definition used in this dataset.

### 3.3 Change in serum albumin (*Δ* albumin)

**Figure 3:**
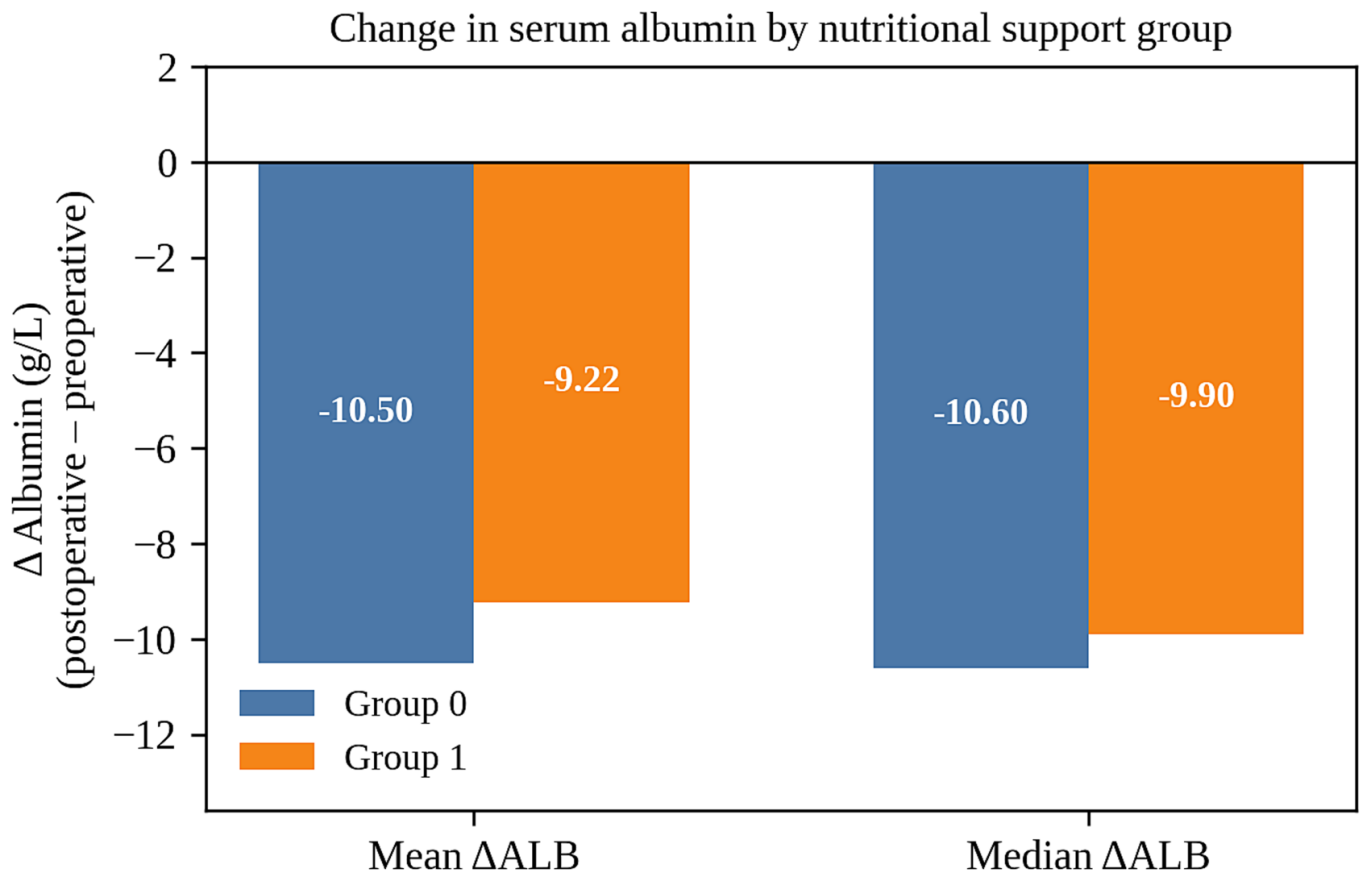
Change in serum albumin (Δ albumin, postoperative minus preoperative; g/L) by nutritional support group (mean and median).

Across the cohort, albumin levels decreased on average in the early postoperative period, consistent with the expected inflammatory and hemodynamic response to major abdominal surgery. Mean *Δ* albumin was −10.50 g/L in Group 0 and −9.22 g/L in Group 1, indicating a smaller postoperative decline among patients who received nutritional support. Median *Δ* albumin values were −10.60 g/L and −9.90 g/L for Group 0 and Group 1, respectively (Figure 3).

### 3.4 Multivariable association analyses

In multivariable linear regression (*n* = 1,297), perioperative nutritional support was associated with a higher Δ albumin (*β* 0.572 g/L; 95% CI 0.111–1.033; *p* = 0.015), corresponding to an attenuated decline in early postoperative albumin after adjustment for prespecified covariates (Table 2). In multivariable logistic regression (*n* = 1,529), nutritional support showed an imprecise association with the binary outcome of albumin improvement (OR 1.331; 95% CI 0.982–1.805; *p* = 0.065) (Table 2). While the point estimate suggested higher odds of albumin improvement among supported patients, the confidence interval included the null, highlighting uncertainty and the potential influence of confounding and exposure heterogeneity.

**Table 2:** Multivariable associations between perioperative nutritional support and albumin recovery outcomes.

Outcome	Model	Effect of nutritional support	95% CI	*p* value	*N*
Δ albumin (g/L)	Multivariable linear regression (HC3 robust SE)	*β* = 0.572	0.111 to 1.033	0.015	1,297
Albumin improvement (binary)	Multivariable logistic regression	OR = 1.331	0.982 to 1.805	0.065	1,529

### 3.5 Prediction model performance and operating characteristics

**Figure 4:**
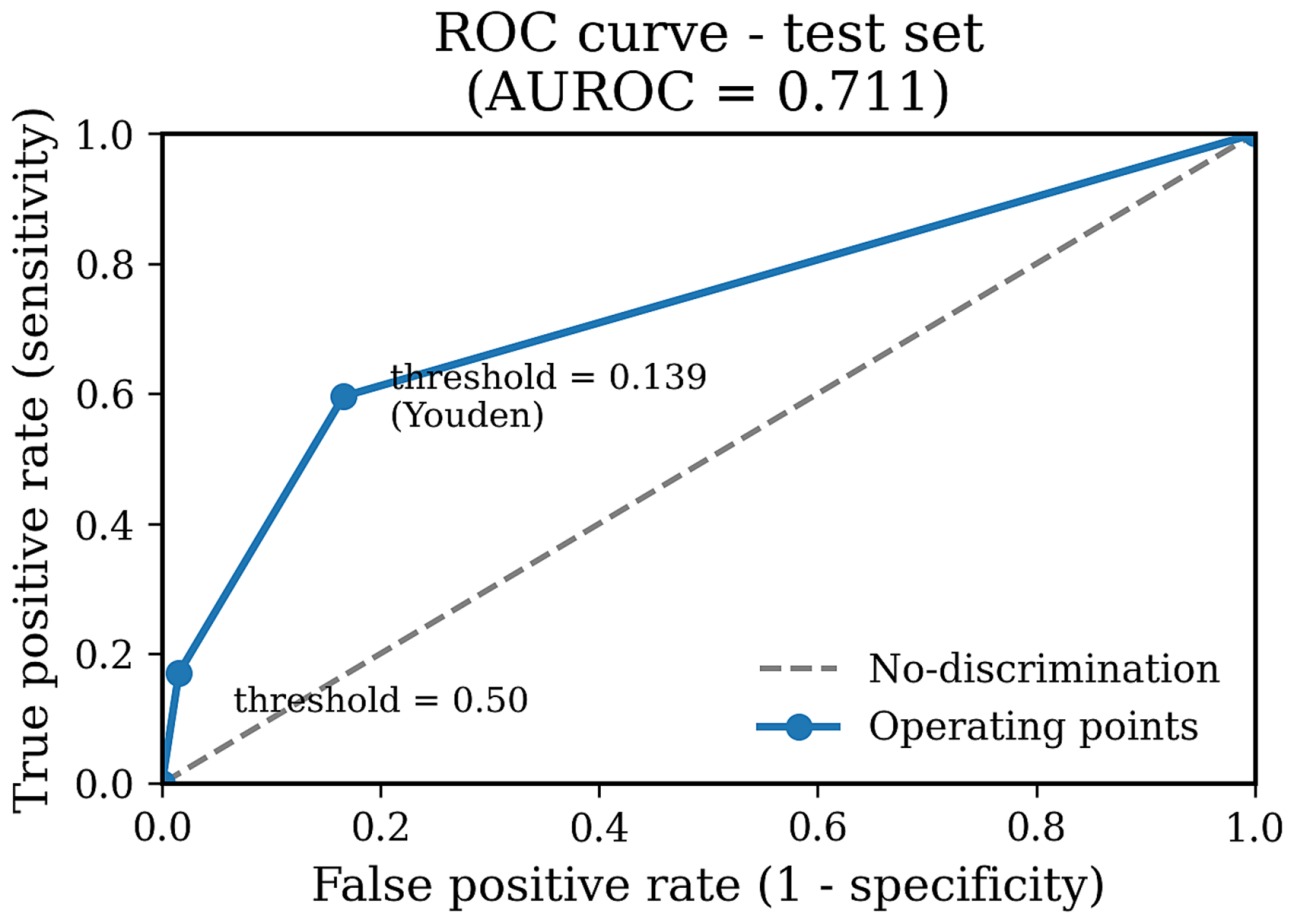
Receiver operating characteristic (ROC) curve for the albumin improvement prediction model in the held-out test set (AUROC = 0.711). Operating points for probability thresholds 0.50 and 0.139 (Youden-index threshold) are indicated.

**Figure 5:**
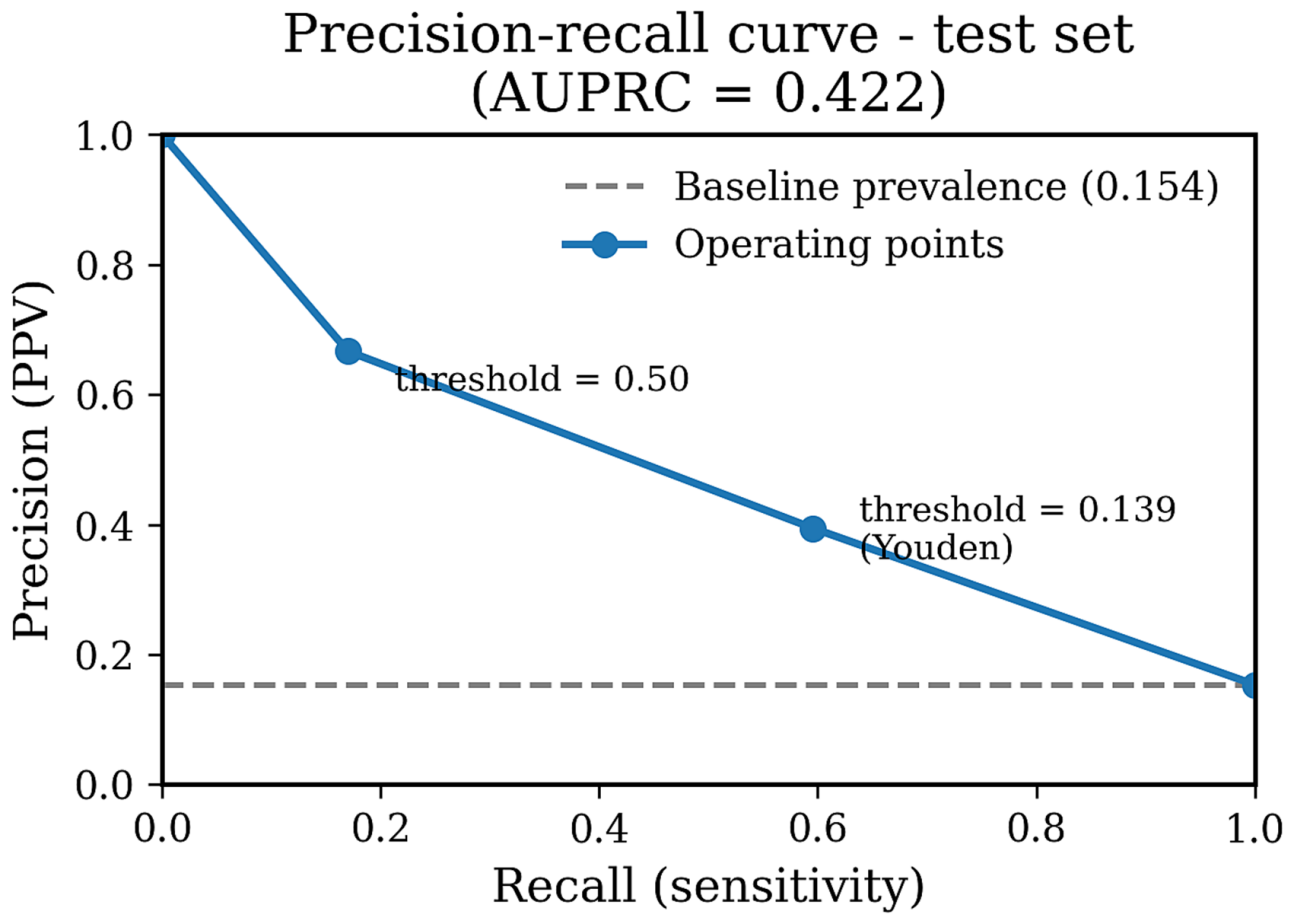
Precision–recall curve for the albumin improvement prediction model in the held-out test set (AUPRC = 0.422). Operating points for probability thresholds 0.50 and 0.139 (Youden-index threshold) are indicated; the dashed line denotes baseline prevalence.

The gradient boosting model demonstrated moderate discrimination for predicting postoperative albumin improvement. In 5-fold cross-validation within the training set, the mean AUROC was 0.683 (SD 0.073) and the mean AUPRC was 0.437 (SD 0.066). On the held-out test set (n = 306), discrimination improved modestly (AUROC 0.711; AUPRC 0.422), and overall calibration error as summarized by the Brier score was 0.1129 (Table 3). ROC and precision–recall curves with highlighted operating points are shown in Figures 4, 5.

**Table 3:** Performance of the albumin improvement prediction model.

Metric	Cross-validation (mean ± SD)	Held-out test set
AUROC	0.683 ± 0.073	0.711
AUPRC	0.437 ± 0.066	0.422
Brier score	Not assessed	0.1129

**Figure 6:**
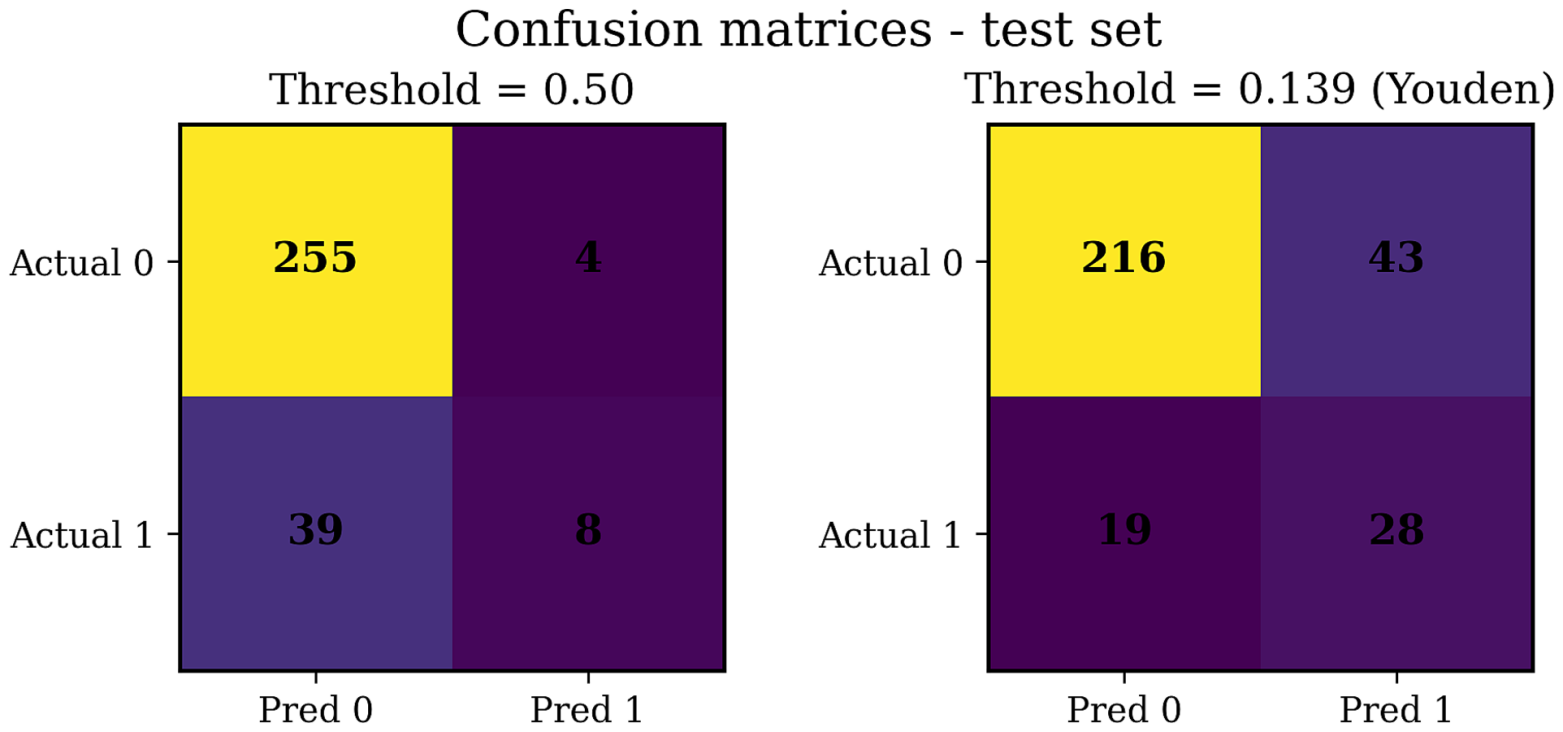
Confusion matrices for the test set at probability thresholds 0.50 and 0.139 (Youden-index threshold).

Threshold selection strongly influenced the clinical trade-off between sensitivity and precision (Table 4; Figure 6). At the conventional 0.50 threshold, the model generated few positive predictions (TP = 8; FP = 4), yielding high precision (0.667) but low recall (0.170). At the lower Youden-index threshold (0.139), recall improved substantially (0.596) with a corresponding decrease in precision (0.394), reflecting more liberal identification of patients predicted to demonstrate albumin improvement. These operating characteristics indicate that the model may be more useful as a screening tool at lower thresholds, whereas higher thresholds prioritize confidence in predicted improvement at the expense of missed cases. At either threshold, the model should be viewed as an adjunct for risk stratification and monitoring, not as a standalone tool for initiating, withholding, or replacing individualized nutritional assessment.

**Table 4:** Threshold-specific classification performance on the test set (*n* = 306).

Threshold	TN	FP	FN	TP	Accuracy	Balanced accuracy	Precision	Recall	F1-score
0.50	255	4	39	8	0.859	0.577	0.667	0.170	0.271
0.139	216	43	19	28	0.797	0.715	0.394	0.596	0.475

### 3.6 Key predictors

**Figure 7:**
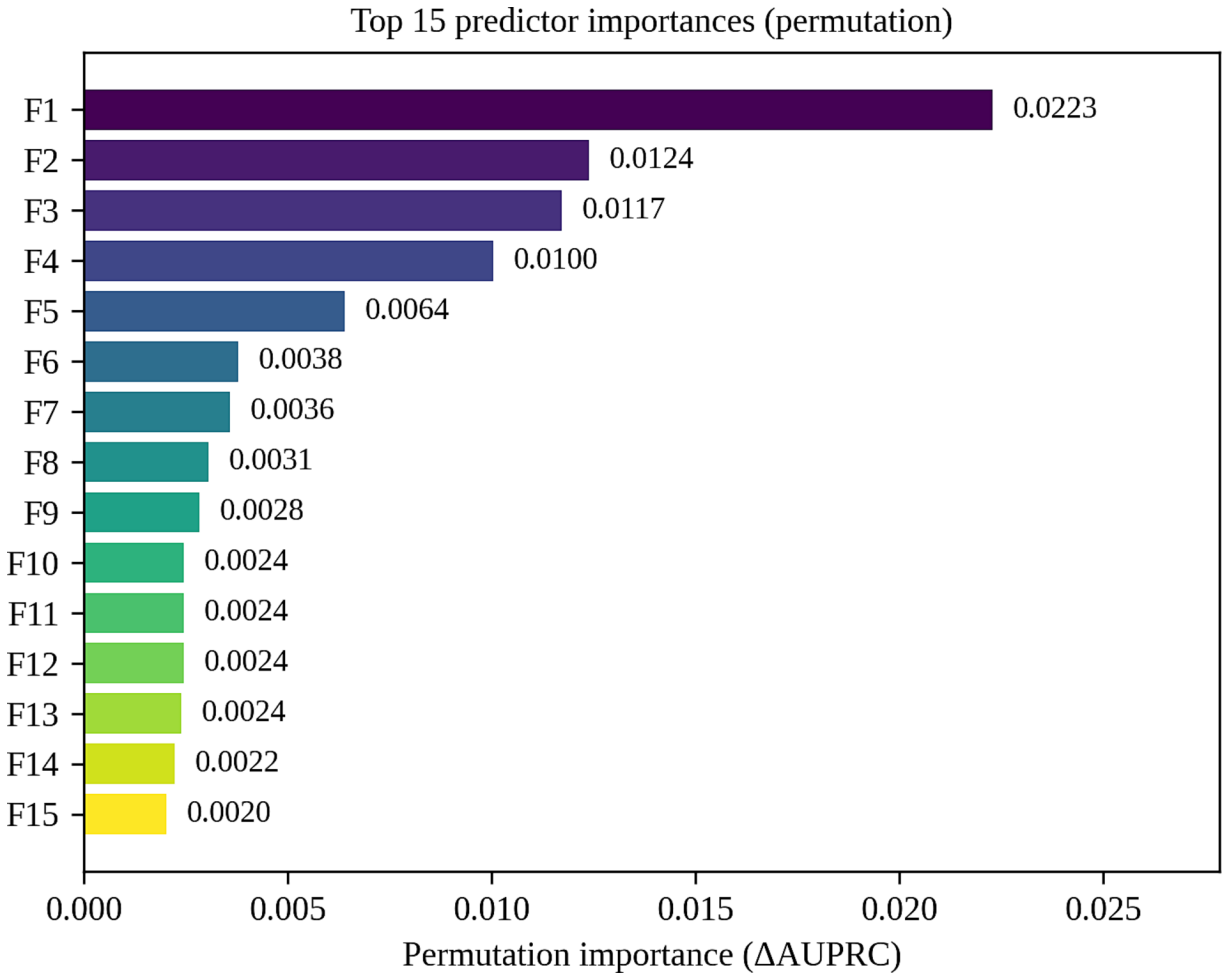
Top 15 predictor importances by permutation importance (*Δ*AUPRC). Feature definitions are provided in Table 5.

Permutation importance analysis identified baseline prealbumin, total protein, and albumin as the most influential predictors, followed by PaCO₂ and several markers reflecting hepatic function and immune status (Table 5; Figure 7). This pattern is clinically plausible because albumin improvement is expected to relate to baseline protein status and systemic physiological reserve, although these predictors may also act as proxies for inflammatory burden and disease severity. Importantly, permutation importance reflects predictive contribution rather than causal relevance, and baseline protein-related variables should be interpreted mainly as markers of underlying condition rather than directly modifiable targets identified by the model.

**Table 5:** Top 15 predictors by permutation importance (scoring metric: AUPRC).

Feature ID	Clinical variable	Permutation importance (ΔAUPRC)
F1	Prealbumin	0.02228
F2	Total protein	0.01239
F3	Albumin	0.01172
F4	PaCO₂ (partial pressure of carbon dioxide)	0.01004
F5	Total bilirubin	0.00639
F6	Lymphocyte count	0.00379
F7	Prothrombin activity (PA)	0.00359
F8	Body mass index	0.00305
F9	Age	0.00284
F10	Neoadjuvant chemotherapy	0.00244
F11	Preoperative total protein	0.00244
F12	White blood cell count	0.00244
F13	Preoperative neutrophil percentage	0.00239
F14	Platelet count	0.00223
F15	Neutrophil count	0.00203

## 4 Discussion

### 4.1 Principal findings

In this retrospective cohort of 1,529 patients undergoing gastrectomy for gastric cancer, perioperative nutritional support was associated with a smaller early postoperative decline in serum albumin (higher *Δ* albumin) and with a higher unadjusted proportion of postoperative albumin improvement. After multivariable adjustment, nutritional support remained positively associated with Δ albumin, whereas the adjusted association with binary albumin improvement was imprecise and did not reach conventional statistical significance. In parallel, a gradient boosting prediction model using routinely collected perioperative variables achieved moderate discrimination for identifying patients likely to show postoperative albumin improvement.

### 4.2 Interpretation in the context of nutritional care pathways

Our findings are consistent with contemporary perioperative nutrition frameworks that emphasize early identification and management of nutritional risk in surgical oncology patients. Guideline-based practice commonly recommends escalation from oral intake optimization to EN and PN when intake is inadequate, particularly in patients with established malnutrition or high nutritional risk. Within this context, the observed association between nutritional support and attenuated albumin decline may reflect improved protein and energy provision and/or better overall perioperative management among patients receiving structured nutritional interventions (2–5). Recent gastrectomy-specific evidence also suggests that a low preoperative cancer cachexia index is associated with severe postoperative morbidity, highlighting that baseline nutritional reserve and cachexia status may shape both the likelihood of receiving perioperative nutritional support and subsequent albumin trajectories (22).

However, the interpretation of serum albumin changes requires caution. Albumin is a negative acute-phase reactant, and early postoperative decreases are driven by a combination of reduced hepatic synthesis, increased capillary permeability, redistribution, and dilution effects. Therefore, an observed attenuation in albumin decline associated with nutritional support may not necessarily indicate improved nutritional status alone; it could also reflect differences in inflammatory burden, perioperative fluid strategies, or unmeasured clinical factors that influence both the likelihood of receiving nutritional support and postoperative albumin kinetics (11, 12). Related work in acute inflammatory surgical disease has shown that thiol-disulfide balance and ischemia-modified albumin can help characterize clinical severity and oxidative stress, reinforcing that albumin-related measures may reflect systemic stress biology as well as nutritional reserve (23). Because C-reactive protein, oxidative-stress biomarkers, detailed fluid balance, surgeon preference, and adherence to individual ERAS components were unavailable in our dataset, residual confounding remains likely.

### 4.3 Binary albumin improvement versus continuous *Δ* albumin

We evaluated both Δ albumin and the binary outcome of albumin improvement to capture complementary aspects of early recovery. Δ albumin retains continuous information about the magnitude of postoperative change and may be more statistically efficient. In contrast, albumin improvement (postoperative > preoperative) is an intuitive endpoint that may be easier to communicate clinically but can be sensitive to measurement timing and analytic dichotomization. The finding that nutritional support was more clearly associated with Δ albumin than with albumin improvement may be explained by information loss from dichotomization, limited event frequency, and the physiologic rarity of an early postoperative albumin increase after major surgery. Dichotomization can also reclassify patients near the threshold because of small differences in assay timing, perioperative fluid administration, or laboratory variability; therefore, the binary endpoint should be regarded as a pragmatic signal of early biochemical trajectory rather than a definitive marker of nutritional recovery.

### 4.4 Comparison with existing literature

Previous studies in gastrointestinal surgery have reported that early postoperative albumin decreases (ΔAlb) are associated with postoperative morbidity, and systematic reviews have supported ΔAlb as a candidate early predictor of complications. In gastrectomy-specific cohorts, postoperative decreases in albumin have been linked to short-term complications even among patients with normal baseline albumin. Our study adds to this literature by focusing on the relationship between perioperative nutritional support and albumin recovery patterns, and by exploring postoperative albumin improvement as an additional recovery indicator (8–10).

Evidence regarding the benefits of perioperative nutritional interventions in gastric cancer surgery is mixed and depends on the type and timing of support. Trials and meta-analyses evaluating oral nutritional supplements and immunonutrition have suggested improvements in selected outcomes, but heterogeneity across interventions and patient populations complicates translation into routine practice (6, 7). In our cohort, nutritional support was associated with a modest attenuation in albumin decline (*β* 0.572 g/L). While this magnitude is small in absolute terms, even modest differences in early biochemical recovery may be clinically meaningful if they reflect broader differences in physiologic stress response or resilience.

### 4.5 Clinical implications

From a clinical standpoint, our findings support an association between perioperative nutritional support and more favorable early postoperative biochemical trajectories, rather than demonstrating that nutritional support directly causes albumin improvement. Nevertheless, because of the retrospective design and potential confounding by indication, these findings should not be interpreted as definitive evidence of a causal effect. Clinicians should continue to follow established nutritional screening and intervention guidelines while recognizing that albumin is an imperfect proxy for nutritional status in the acute postoperative period (2–5).

The lack of a clear adjusted association with the binary albumin improvement outcome also suggests that albumin improvement, as defined here, may be too stringent or may depend critically on the timing of postoperative measurement. Future work should evaluate alternative, clinically motivated endpoints such as (i) absolute *Δ* albumin thresholds, (ii) percentage change in albumin, (iii) albumin recovery trajectories across multiple postoperative days, and (iv) composite endpoints that incorporate inflammatory markers, functional recovery, and complication rates.

### 4.6 Implications for prediction modeling and model deployment

The prediction model achieved moderate discrimination (test AUROC 0.711; test AUPRC 0.422). Given the outcome prevalence (approximately 15%), the AUPRC indicates performance above the baseline prevalence but also underscores that many positive predictions will be false positives at thresholds designed for higher sensitivity. Therefore, a key consideration is the intended use case. A lower threshold (e.g., 0.139) may be appropriate for screening and prioritization of nutritional assessment or closer monitoring, whereas higher thresholds may be reserved for situations where resources are scarce and high precision is needed. In practice, a low threshold could flag patients for dietitian review, closer laboratory surveillance, or complication monitoring, whereas a high threshold could prioritize patients most likely to show the endpoint; no threshold should be used to withhold clinically indicated nutritional support. The model is intended to support, not replace, clinician judgment. Its value lies in a disease-specific, structured perioperative prediction task, which differs from broad general-purpose AI systems; recent evidence that large language models underperform human experts and residents on European general surgery board-style examinations reinforces the need for specialized, transparently validated clinical decision-support models (24).

For clinical translation, additional steps are required. First, external validation in independent cohorts is essential to evaluate generalizability and recalibrate predicted probabilities across settings. Second, the model should be assessed for potential bias and differential performance across clinically relevant subgroups (e.g., age strata, tumor stage, baseline nutritional risk). Third, prospective impact studies are needed to determine whether prediction-guided nutritional strategies improve patient-centered outcomes such as complication rates, quality of recovery, and length of stay (14–17). Because cross-validation and the 80/20 test split were performed within the same single-center 2017 cohort, validation remains internal only and apparent performance may be optimistic when applied to other hospitals, time periods, or nutrition-support protocols.

### 4.7 Strengths and limitations

This study has several strengths. The cohort size was relatively large for a single disease-specific surgical population, and we evaluated both association and prediction objectives using clinically accessible endpoints. The prediction model used routinely available variables and was evaluated using metrics appropriate for imbalanced outcomes, with transparent reporting of operating points.

Limitations should be acknowledged. First, the observational design is susceptible to confounding by indication; patients who received nutritional support may have differed systematically from those who did not. For example, patients with overt nutritional risk may have been more likely to receive support, whereas patients able to tolerate oral or enteral intake may have differed in ways not captured by the dataset. Second, the exposure was represented as a binary classification and may not capture the heterogeneity of nutritional support modality, dose, timing, and adherence. Third, albumin is influenced by inflammation and perioperative fluid management, and the timing of postoperative measurement may vary, which can introduce measurement variability. Fourth, the multivariable linear regression was conducted in a complete-case sample, which may limit representativeness if missingness is not completely at random. Finally, the prediction model was internally validated only; without external validation, performance estimates may be optimistic for other clinical settings. In addition, because the cohort was derived from 2017 records, contemporary patient presentations may differ. Pandemic-era data suggest that healthcare disruptions can be associated with delayed diagnosis, more advanced gastric cancer, and lower BMI or altered nutritional status, which may affect the baseline distribution of the 46 candidate predictors in modern cohorts (25). Complete-case analysis excluded 232 patients from the *Δ* albumin regression, and bias is possible if laboratory missingness reflected illness severity or treatment pathways rather than random administrative factors.

### 4.8 Future directions

Future research should (i) refine exposure definitions by capturing the type, duration, and caloric/protein targets of nutritional support; (ii) incorporate perioperative inflammatory markers and fluid balance measures to better contextualize albumin kinetics; (iii) evaluate albumin recovery trajectories across multiple postoperative timepoints; and (iv) perform external validation and prospective evaluations of prediction-guided nutritional interventions. Methodologically, causal inference approaches such as propensity score methods or target trial emulation may help reduce confounding and clarify whether nutritional support contributes causally to improved recovery markers.

## 5 Conclusion

In this retrospective cohort of patients undergoing gastrectomy for gastric cancer, perioperative nutritional support was associated with an attenuated early postoperative decline in serum albumin (higher *Δ* albumin). A gradient boosting model using routine perioperative variables provided moderate discrimination for predicting postoperative albumin improvement. External validation and prospective studies are needed to clarify causality and to determine whether prediction-guided nutritional strategies improve clinically meaningful outcomes.

## Data Availability

The original contributions presented in the study are included in the article/supplementary material, further inquiries can be directed to the corresponding authors.
